# A review of functionality assessment scales in schizophrenia

**DOI:** 10.3389/fpsyt.2025.1640963

**Published:** 2025-12-16

**Authors:** Petru Ifteni, Ana Aliana Miron, Paula Simina Petric, Andreea Teodorescu, Mihnea Costin Manea

**Affiliations:** 1 Transilvania University of Brașov, Brașov, Romania; 2 Clinical Hospital of Psychiatry and Neurology, Brașov, Romania; 3 University of Medicine and Pharmacy “Carol Davila”, Bucharest, Romania; 4 Clinical Psychiatric Hospital “Prof. Dr. Alexandru Obregia”, Bucharest, Romania

**Keywords:** functionality, schizophrenia, scale, outcome, disability

## Abstract

**Introduction:**

Schizophrenia is a severe and chronic psychiatric disorder marked by a complex and heterogeneous array of symptoms, including positive symptoms (such as hallucinations and delusions), negative symptoms (such as emotional blunting and social withdrawal), and cognitive deficits. These symptoms result in profound and persistent impairments across multiple domains of functioning, including self-care, interpersonal relationships, family life, and occupational performance. Therefore, evaluation of functionality in schizophrenia has become an important objective for monitoring the clinical and therapeutic outcome of the patients.

**Methods:**

This review identifies and evaluates the development and use of functioning assessment scales in schizophrenia from 1976 to 2024. A comprehensive search of four major medical databases (PubMed, NIH, Wiley Online Library, and Springer Nature Link) covering studies published over nearly five decades yielded 42 distinct instruments that met predefined inclusion criteria.

**Results:**

Each scale was examined in terms of its clinical utility, psychometric robustness, domains assessed, and the extent to which it integrates the perspectives of patients, clinicians, and caregivers. The analysis revealed a wide variation in the domains covered by these tools, many focusing heavily on either clinical or social aspects of functioning, often neglecting others such as occupational or familial roles. Additionally, the usability of several instruments in routine clinical settings was limited by their length or complexity.

**Discussions:**

These findings underscore the need for more streamlined, multidimensional, and user-friendly assessment tools that are both scientifically rigorous and practically applicable. The review highlights the importance of adopting a holistic approach to functional recovery in schizophrenia—one that considers not only symptom reduction but also quality of life and reintegration into the community. Future scale development should prioritize the inclusion of input from all key stakeholders and aim to capture the nuanced realities of daily functioning in individuals living with schizophrenia.

## 1 Introduction

Schizophrenia has been defined as a severe mental disorder, affecting approximately 24 million people or 1 in 300 people worldwide (1). It follows a chronic and heterogeneous course, significantly impairing quality of life. Hallmark features include hallucinations, disorganized speech and behavior, along with other symptoms that lead to social or occupational dysfunction, such as cognitive, attentional, and memory deficits (2). The incidence is estimated at 15.2 cases per 100,000 people. Onset occurs between the ages of 12 and 25, placing considerable pressure on healthcare systems and generating substantial economic costs for both individuals and society (3). Another peak of onset age is 30 to 35 years (4). Schizophrenia typically begins during adolescence or early adulthood, and affects individuals experience an early loss of their capacity for social and occupational functioning, often before having stable careers or social networks. Early onset is associated with a severe disease course, functional impairment, and a poorer prognosis in terms of long-term socio-professional integration (5).

Schizophrenia is one of the most disabling psychiatric disorders and ranks among the top 20 causes of disability worldwide, along with depression, alcohol use disorders, and other psychoses (6). While positive symptoms are the core of the diagnosis, functional deficits constitute a major component of the illness. Decline in autonomous functioning is a defining feature and can be predictive of disease trajectory (7).

In the context of functional impairment in schizophrenia, “functionality” refers to the ability of an individual to perform daily activities and live independently, encompassing domains such as self-care, social interaction, interpersonal relationships, employment, and management of everyday tasks. These areas are interdependent, with deficits in one domain potentially exacerbating dysfunction in others (8).

Although schizophrenia impairs functioning across these areas, outcomes may vary. A small subset of patients achieves normative social functioning comparable to that of healthy individuals. However, the majority demonstrate intermediate levels of functionality, while others exhibit profound deficits and severe disability (9).

The functional impact of schizophrenia varies significantly depending on the severity of psychotic symptoms, absence of treatment, general health status, environmental factors, social cognition, and sociodemographic variables (10). Psychosis can severely affect a patient’s ability to organize and make judgments, while negative symptoms such as anhedonia, social withdrawal, and avolition can substantially reduce everyday functioning and autonomy (11). Cognitive deficits, including impaired processing speed, attention, memory, verbal communication, and reasoning, may also contribute to impaired daily functioning. Social cognition, in particular, is closely associated with overall functionality in schizophrenia (12). For example, in the study of Minglan Yu et al., patients with more than six months of untreated psychosis exhibited more severe negative symptoms and greater cognitive and social decline compared to those treated within six months of symptom onset (13).

Patients with schizophrenia have higher rates of obesity and associated comorbidities (e.g., cardiovascular disease, hypertension) than the general population (14). Obesity-related impairments in mobility, flexibility, coordination, and gait efficiency often hinder basic activities such as dressing, bathing, climbing stairs, housework, or shopping, therefore leading to a negative impact on functionality. Additionally, obesity can also negatively impact cognition. Consequently, obesity is an important factor that contributes to functional impairment (15).

All disease-associated factors contribute to a pervasive impairment of individuals with schizophrenia, increased morbidity and mortality rates, and a substantially reduced life expectancy compared to the general population (16).

Environmental factors such as financial instability, living in disadvantaged communities, and unemployment also contribute to functional impairment in patients with schizophrenia (17). Demographic factors, including higher education levels, female gender, and employment history, are associated with better functional outcomes (18).

While long-term societal integration (e.g., employment retention) remains a key treatment goal, such outcomes often exceed the scope or time frame of clinical trials for novel antipsychotic agents. Consequently, a wide range of instruments has been developed to evaluate functional outcomes in schizophrenia. These instruments can be broadly categorized into performance-based and interview-based measures.

Interview-based instruments include: (1) clinician-rated interview scales, based on interviews with the patient and/or caregiver, assessing frequency and quality of participation in social, occupational, and academic activities, and (2) self-report measures, assessing subjective experiences of functioning, which often overlap with quality of life.

Performance-based instruments are functional capacity measures, and they evaluate clinician assessments of the patient’s ability to perform daily tasks and performance under controlled conditions, or the assessment of the actual ability to carry out tasks in a standardized environment (19).

Clinician-rated interview scales are widely used in schizophrenia for evaluating functional status. They provide general information but have limited sensitivity for specific subdomains (e.g., social or occupational functioning). Their administration requires clinical expertise and training to ensure accuracy. A further limitation is the lack of detailed grading criteria, which may raise concerns about validity (20). Moreover, some patients may be unable or unwilling to accurately report their behavior and activities, though experienced clinicians with ongoing patient contact may mitigate these issues. Alternatively, information can be obtained from a caregiver or social worker familiar with the case (21).

Certain standardized instruments are structured as self-report scales, wherein patients rate their own daily functioning. However, such self-assessments correlate weakly with clinician-rated or performance-based measures, suggesting they offer distinct perspectives on the therapeutic process (19).

Functional capacity assessment instruments measure the ability to perform tasks under controlled conditions, instead of measuring these abilities in an unsupervised environment. Improvement in functional capacity is considered an important intermediate outcome facilitating behavioral change. These assessments have been shown to mediate the relationship between cognitive ability and real-world functioning (8). They provide insight into specific deficits and can inform targeted interventions. Nevertheless, capacity does not always translate into functional performance in naturalistic settings (19).

In the late years, the approach to treating schizophrenia has evolved: functional recovery is now a key therapeutic goal. Improving real-world functioning is viewed as essential, surpassing simple symptom reduction, because it directly supports reintegration and enhances quality of life. As a result, thorough and accurate evaluation of patient functioning has become crucial for both clinical monitoring and treatment planning. It is therefore important to review the existing functionality assessment tools, particularly from a practical and clinical perspective.

The Global Assessment of Functioning Scale (GAFS) was the first standardized instrument used to assess functionality in schizophrenia. Introduced in 1976 by Jane Endicott and colleagues, it scores overall functioning on a scale from 0 to 100, considering psychological symptoms and functioning across personal, occupational, and social domains. Although easy to use, its limitations include subjectivity and a lack of domain-specific scores. GAFS use declined after the introduction of DSM-5 in 2013, as other instruments gained prominence (22).

Currently, the Personal and Social Performance Scale (PSP) is the most widely used instrument (23). It distinguishes four functional domains: social relationships, daily activities and self-care, and aggressive behavior. PSP uses a 0–100 scoring system, with 0 indicating severe dysfunction and 100 representing normal functioning. Its advantages include specificity for schizophrenia, the ability to detect functional change over time, and domain granularity. Limitations include clinician subjectivity and the need for training to ensure reliable application (23).

## 2 Aims

The primary objective of this paper was to identify the main functionality assessment scales developed and applied to patients with schizophrenia between 1976 and 2024, and to analyze the functional domains assessed by each scale—namely, personal, social, familial, and occupational functioning—in the context of their clinical utility. We aimed to review the existing scales for functionality assessment in schizophrenia, mainly, but not only, from the clinicians’ perspective, and focusing on practical and applicability features.

## 3 Materials and methods

### 3.1 Article screening

For the purpose of this systematic review, articles focusing on the evaluation of functionality scales in schizophrenia, published between 01 January 1976 and 31 December 2024, were identified from the scientific literature. The search was focused on scale development articles and validation studies for functionality assessment scales in schizophrenia patients. The review protocol was conducted according to PRISMA Guidelines. The protocol was not registered in PROSPERO. There was no assessment for risk of bias or specific method to synthesize results.

The following electronic databases were used: PubMed, NIH, Wiley Online Library, and Springer Nature Link. First search was performed on 10 December 2024 and last search on 10 May 2025. The search strategy incorporated keywords such as: *“functionality”, “schizophrenia”, “scale”, “impairment”, “social”, “occupational”, “professional”, “outcome”*, or *“disability”.* Boolean operators “AND” and “OR” were also used for the search, in various combinations: “functionality AND schizophrenia AND scale”, “disability AND scale AND schizophrenia”, “social OR occupational AND impairment AND schizophrenia”, “social OR professional AND outcome AND schizophrenia”, “functionality AND impairment AND schizophrenia”, “social AND ocupational AND disability AND schizophrenia”. Title and full-text screening were conducted in duplicate and independently by two reviewers (TA, PPS, MMC). Disagreements were resolved through consensus mediated by a third author (IP, MAA).Inclusion criteria for the database construction were: (1) full-text studies, (2) studies published in English, (3) studies published between 1976 and 2024, and (4) studies specifically addressing functionality assessment scales in patients diagnosed with schizophrenia. Exclusion criteria were: (1) studies published in languages other than English, (2) studies published prior to 1976, (3) duplicate entries, (4) incomplete studies, and (5) studies focusing on other psychiatric disorders.

### 3.2 Data extraction

Data extraction was conducted independently by two of the authors using a Microsoft Excel spreadsheet. For each article analyzed, the following information was collected: author, publication year, publication DOI, instrument/scale title, rater type (physician, caregiver, patient), estimated assessment time, and assessed domains (**Table 1**).

**Table 1:** Analyzed articles.

No.	Authors	Scale	Rater type	Relative administration time (minutes)	Citation
1	Rybarczyk, B.	Social and Occupational Functioning Assessment Scale (SOFAS)	Physician	5	Rybarczyk, B. (2018). Social and occupational functioning assessment scale (SOFAS). In *Encyclopedia of Clinical Neuropsychology* (pp. 1–1). Springer International Publishing.
2	Morosini, P. L., Magliano, L., Brambilla, L., Ugolini, S., & Pioli, R.	Personal and Social Performance Scale (PSP)	Physician	20	Morosini, P. L., Magliano, L., Brambilla, L., Ugolini, S., & Pioli, R. (2000). Development, reliability and acceptability of a new version of the DSM-IV Social and Occupational Functioning Assessment Scale (SOFAS) to assess routine social functioning. *Acta psychiatrica Scandinavica*, *101*(4), 323–329.
3	Wiersma, D., DeJong, A., & Ormel, J.	Groningen Social Disabilities Schedule (GSDS-II)	Physician	40	Wiersma, D., DeJong, A., & Ormel, J. (1988). The Groningen Social Disabilities Schedule: development, relationship with I.C.I.D.H., and psychometric properties. *International Journal of Rehabilitation Research. Internationale Zeitschrift Für Rehabilitationsforschung. Revue Internationale de Recherches de Readaptation*, *11*(3), 213–224.
4	Wallace CJ, Liberman RP, Tauber R, Wallace J	Independent Living Skills Survey (ILSS)	Caregiver/Patient	35/25	Wallace CJ, Liberman RP, Tauber R, Wallace J. The independent living skills survey: a comprehensive measure of the community functioning of severely and persistently mentally ill individuals. Schizophr Bull. 2000;26(3):631-58
5	Rosen, A., Hadzi-Pavlovic, & D., Parker, G.	Abbreviated Life Skills Profile (LSP-16)	Physician	10	Rosen, A., Hadzi-Pavlovic, & D., Parker, G. (1989). The Life Skills Profile: A measure assessing function and disability in schizophrenia. *Schizophrenia Bulletin*. 15(2): 325-337.
6	Luján-Lujan EM, García-León MÁ, Rodriguez-Cano E, Huertas-Martínez S, Roldan-Merino J, Puig-Llobet M, Miguel-Ruiz MD, Salvador R, Vieta E, Pomarol-Clotet E.	Functioning Assessment Short Test (FAST)	Physician	15	Luján-Lujan EM, García-León MÁ, Rodriguez-Cano E, Huertas-Martínez S, Roldan-Merino J, Puig-Llobet M, Miguel-Ruiz MD, Salvador R, Vieta E, Pomarol-Clotet E. (2022). Validity of the Functioning Assessment Short Tests (FAST), in patients with schizophrenia. Rev Psiquiatr Salud Ment (Engl Ed). 15(3):157-166.
7	Barker, S., Barron, N., McFarland, B. H., & Bigelow, D. A.	Multnomah Community Ability Scale (MCAS)	Physician	15	Barker, S., Barron, N., McFarland, B. H., & Bigelow, D. A. (1994). A community ability scale for chronically mentally ill consumers: Part I. Reliability and validity. *Community Mental Health Journal*, *30*(4), 363–383.
8	Burckhardt, C. S., & Anderson, K. L.	The Quality of Life Scale (QOLS)	Patient	5	Burckhardt, C. S., & Anderson, K. L. (2003). The Quality of Life Scale (QOLS): reliability, validity, and utilization. *Health and Quality of Life Outcomes*, *1*(1), 60.
9	Heinrichs, D. W., Hanlon, T. E., & Carpenter, W. T., Jr.	Quality of Life Scale (QOL Heinrichs)	Physician	45	Heinrichs, D. W., Hanlon, T. E., & Carpenter, W. T., Jr. (1984). The Quality of Life Scale: an instrument for rating the schizophrenic deficit syndrome. *Schizophrenia Bulletin*, *10*(3), 388–398.
10	Endicott, J., Spitzer, R. L., Fleiss, J. L., & Cohen, J.	Global Assessment of Functioning (GAF)	Physician	5	Endicott, J., Spitzer, R. L., Fleiss, J. L., & Cohen, J. (1976). The global assessment scale. A procedure for measuring overall severity of psychiatric disturbance. *Archives of General Psychiatry*, *33*(6), 766–771.
11	Patterson, T. L., Goldman, S., McKibbin, C. L., Hughs, T., & Jeste, D. V.	University of California San Diego Performance-Based Skills Assessment (UPSA)	Physician	30	Patterson, T. L., Goldman, S., McKibbin, C. L., Hughs, T., & Jeste, D. V. (2001). UCSD Performance-Based Skills Assessment: development of a new measure of everyday functioning for severely mentally ill adults. *Schizophrenia Bulletin*, *27*(2), 235–245.
12	Revheim, N.& Medalia, A.	Independent Living Scales (ILS)	Physician	55	Revheim N, Medalia A. (2004). The independent living scales as a measure of functional outcome for schizophrenia. Psychiatr Serv.;55(9):1052-4.
13	Velligan, D. I., Fredrick, M., Mintz, J., Li, X., Rubin, M., Dube, S., Deshpande, S. N., Trivedi, J. K., Gautam, S., Avasthi, A., Kern, R. S., & Marder, S. R.	Test of Adaptive Behavior in Schizophrenia (TABS)	Physician	40	Velligan, D. I., Fredrick, M., Mintz, J., Li, X., Rubin, M., Dube, S., Deshpande, S. N., Trivedi, J. K., Gautam, S., Avasthi, A., Kern, R. S., & Marder, S. R. (2014). The reliability and validity of the MATRICS functional assessment battery. *Schizophrenia Bulletin*, *40*(5), 1047–1052.
14	Ruse, S. A., Davis, V. G., Atkins, A. S., Krishnan, K. R. R., Fox, K. H., Harvey, P. D., & Keefe, R. S. E.	The Virtual Reality Functional Capacity Assessment Tool (VRFCAT)	Physician	30	Ruse, S. A., Davis, V. G., Atkins, A. S., Krishnan, K. R. R., Fox, K. H., Harvey, P. D., & Keefe, R. S. E. (2014). Development of a virtual reality assessment of everyday living skills. *Journal of Visualized Experiments: JoVE*, *86*.
15	Potkin, S., Bugarski-Kirola, D., Edgar, C., & Luo, S.	Readiness for Work Questionnaire (WoRQ)	Physician	5	Potkin, S., Bugarski-Kirola, D., Edgar, C., & Luo, S. (2012). PRM30 evaluating readiness for work in patients with schizophrenia: “the readiness for work questionnaire” (WoRQ). *Value in Health: The Journal of the International Society for Pharmacoeconomics and Outcomes Research*, *15*(7), A650.
16	Barak, Y., & Aizenberg, D.	Psychosocial Remission in Schizophrenia Scale (PSRS)	Physician	15	Barak, Y., & Aizenberg, D. (2012). Clinical and psychosocial remission in schizophrenia: correlations with antipsychotic treatment. *BMC Psychiatry*, *12*, 108.
17	Mausbach, B. T., Harvey, P. D., Goldman, S. R., Jeste, D. V., & Patterson, T. L.	Performance-Based Skills Assessment-Brief Version (UPSA-B)	Physician	5	Mausbach, B. T., Harvey, P. D., Goldman, S. R., Jeste, D. V., & Patterson, T. L. (2007). Development of a brief scale of everyday functioning in persons with serious mental illness. *Schizophrenia Bulletin*, *33*(6), 1364–1372.
18	Lawton, M. P., & Brody, E. M.	The Lawton Instrumental Activities of Daily Living Scale (LIADL)	Physician	15	Lawton, M. P., & Brody, E. M. (1969). Assessment of older people: Self-maintaining and instrumental activities of daily living. *The Gerontologist*, *9*(3 Part 1), 179–186.
19	Ware, J. E., & Sherbourne, C. D.	Short form-36 Health Survey (SF-36)	Patient	10	Ware, J. E., & Sherbourne, C. D. (1992). The MOS 36-ltem short-form health survey (SF-36): I. conceptual framework and item selection. *Medical Care*, *30*(6), 473–483.
20	Lancon C, Auquier P, Launois R, Toumi M, Llorca PM, Bebbington P, Lehman A	Quality of life interview (QoLI)	Physician	16	Lancon C, Auquier P, Launois R, Toumi M, Llorca PM, Bebbington P, Lehman A. (2000). Evaluation of the quality of life of schizophrenic patients: validation of the brief version of the Quality of Life Interview. Encephale;26(4):11-6
21	Oliver JP, Huxley PJ, Priebe S, Kaiser W	The Lancashire Quality of Life Profile (LQOLP)	Physician	35	Oliver JP, Huxley PJ, Priebe S, Kaiser W. (1997). Measuring the quality of life of severely mentally ill people using the Lancashire Quality of Life Profile. Soc Psychiatry Psychiatr Epidemiol.;32(2):76-83
22	Kleinman, L., Lieberman, J., Dube, S., Mohs, R., Zhao, Y., Kinon, B., Carpenter, W., Harvey, P. D., Green, M. F., Keefe, R. S. E., Frank, L., Bowman, L., & Revicki, D. A.	The Schizophrenia Outcomes Functioning Interview (SOFI)	Physician	45	Kleinman, L., Lieberman, J., Dube, S., Mohs, R., Zhao, Y., Kinon, B., Carpenter, W., Harvey, P. D., Green, M. F., Keefe, R. S. E., Frank, L., Bowman, L., & Revicki, D. A. (2009). Development and psychometric performance of the schizophrenia objective functioning instrument: an interviewer administered measure of function. *Schizophrenia Research*, *107*(2–3), 275–285
23	Schneider, L. C., & Struening, E. L.	Specific Level of Functioning Scale (SLOF)	Caregiver	15	Schneider, L. C., & Struening, E. L. (2016). Specific level of functioning scale. In *PsycTESTS Dataset*. American Psychological Association (APA).
24	Llorca, P.-M., Lançon, C., Lancrenon, S., Bayle, F.-J., Caci, H., Rouillon, F., & Gorwood, P.	Functional Remission of General Schizophrenia (FROGS)	Physician	30	Llorca, P.-M., Lançon, C., Lancrenon, S., Bayle, F.-J., Caci, H., Rouillon, F., & Gorwood, P. (2009). The “Functional Remission of General Schizophrenia” (FROGS) scale: development and validation of a new questionnaire. *Schizophrenia Research*, *113*(2–3), 218–225.
25	Alonso J, Olivares J, Ciudad A, Manresa J, Casado A, Gilaberte I.	Social Functioning Scale (SFS)- short version	Physician	15	Alonso J, Olivares J, Ciudad A, Manresa J, Casado A, Gilaberte I. (2008). Development and validation of the Social Functioning Scale, short version, in schizophrenia for its use in the clinical practice. Actas Esp Psiquiatr.;36(2):102-10.
26	Pukrop, R., Möller, H. J., & Steinmeyer, E. M.	Modular System for Quality of Life (MSQL-R)	Patient	60	Pukrop, R., Möller, H. J., & Steinmeyer, E. M. (2000). Quality of life in psychiatry: a systematic contribution to construct validation and the development of the integrative assessment tool “modular system for quality of life.” *European Archives of Psychiatry and Clinical Neuroscience*, *250*(3), 120–132.
27	Tyrer, P., Nur, U., Crawford, M., Karlsen, S., McLean, C., Rao, B., & Johnson, T.	Social Functioning Questionnaire (SFQ)	Patient	5	Tyrer, P., Nur, U., Crawford, M., Karlsen, S., McLean, C., Rao, B., & Johnson, T. (2011). Social Functioning Questionnaire. In *PsycTESTS Dataset*. American Psychological Association (APA).
28	Ritsner, M., Kurs, R., Gibel, A., Ratner, Y., & Endicott, J.	Quality of Life Enjoyment and Satisfaction Questionnaire - 18-item (Q-LES-Q-18)	Patient	10	Ritsner, M., Kurs, R., Gibel, A., Ratner, Y., & Endicott, J. (2005). Validity of an abbreviated quality of life enjoyment and satisfaction questionnaire (Q-LES-Q-18) for schizophrenia, schizoaffective, and mood disorder patients. *Quality of Life Research: An International Journal of Quality of Life Aspects of Treatment, Care and Rehabilitation*, *14*(7), 1693–1703.
29	Takahashi, M., Tanaka, K., & Miyaoka, H.	Communication Skills Questionnaire (CSQ)	Patient	10	Takahashi, M., Tanaka, K., & Miyaoka, H. (2006). Reliability and validity of communication skills questionnaire (CSQ). *Psychiatry and Clinical Neurosciences*, *60*(2), 211–218.
30	Cooper, P., Osborn, M., Gath, D., & Feggetter, G.	Social Adjustment Scale Modified (SAS-M)	Patient	15	Cooper, P., Osborn, M., Gath, D., & Feggetter, G. (1982). Evaluation of a modified self-report measure of social adjustment. *The British Journal of Psychiatry: The Journal of Mental Science*, *141*(1), 68–75.
31	Roldán-Merino, J., Lluch-Canut, T., Menarguez-Alcaina, M., Foix-Sanjuan, A., Haro Abad, J. M., & QuestERA Working Group.	Self-Care Requisites Scale (ERA)	Patient	30	Roldán-Merino, J., Lluch-Canut, T., Menarguez-Alcaina, M., Foix-Sanjuan, A., Haro Abad, J. M., & QuestERA Working Group. (2014). Psychometric evaluation of a new instrument in Spanish to measure self-care requisites in patients with schizophrenia: Psychometric evaluation of a new instrument in Spanish to measure self-care requisites in patients with schizophrenia. *Perspectives in Psychiatric Care*, *50*(2), 93–101.
32	Boyer, L., Simeoni, M.-C., Loundou, A., D’Amato, T., Reine, G., Lancon, C., & Auquier, P.	Schizophrenia Quality of Life Questionnaire Short Form – Clinical Practice (S-QOL 18)	Patient	5	Boyer, L., Simeoni, M.-C., Loundou, A., D’Amato, T., Reine, G., Lancon, C., & Auquier, P. (2010). The development of the S-QoL 18: a shortened quality of life questionnaire for patients with schizophrenia. *Schizophrenia Research*, *121*(1–3), 241–250.
33	Su, C.-T., Yang, A.-L., & Lin, C.-Y.	Schizophrenia Quality of Life Scale Revision 4 (SQLS-R4)	Patient	10	Su, C.-T., Yang, A.-L., & Lin, C.-Y. (2017). The construct of the Schizophrenia Quality of Life Scale Revision 4 for the population of Taiwan. *Occupational Therapy International.*
34	Yildiz, M., Erim, R., Soygur, H., Tural, U., Kiras, F., & Gules, E.	Subjective Recovery Assessment Scale (SubRAS)	Patient	10	Yildiz, M., Erim, R., Soygur, H., Tural, U., Kiras, F., & Gules, E. (2018). Development and validation of the Subjective Recovery Assessment Scale for patients with schizophrenia. *Psychiatry and Clinical Psychopharmacology*, *28*(2), 163–169.
35	Cale, E. L., Deane, F. P., Kelly, P. J., & Lyons, G. C. B.	Recovery Assessment Scale – 24 items (RAS-24)	Patient	15	Cale, E. L., Deane, F. P., Kelly, P. J., & Lyons, G. C. B. (2015). Psychometric properties of the Recovery Assessment Scale in a sample with substance use disorder. *Addiction Research & Theory*, *23*(1), 71–80.
36	He, S.-J., Fang, Y.-W., Huang, Z.-X., & Yu, Y.	Recovery Assessment Scale – 8 items (RAS-8)	Patient	5	He, S.-J., Fang, Y.-W., Huang, Z.-X., & Yu, Y. (2021). Validation of an 8-item Recovery Assessment Scale (RAS-8) for people with schizophrenia in China. *Health and Quality of Life Outcomes*, *19*(1), 119.
37	Carlson, J., Ochoa, S., Haro, J. M., Escartín, G., Ahuir, M., Gutierrez-Zotes, A., Salamero, M., Valero, J., Cañizares, S., Bernardo, M., Cañete, J., & Gallo, P.	Satisfaction with Life Domains Scale (SLDS)	Patient	15	Carlson, J., Ochoa, S., Haro, J. M., Escartín, G., Ahuir, M., Gutierrez-Zotes, A., Salamero, M., Valero, J., Cañizares, S., Bernardo, M., Cañete, J., & Gallo, P. (2009). Adaptation and validation of the quality-of-life scale: Satisfaction with Life Domains Scale by Baker and Intagliata. *Comprehensive Psychiatry*, *50*(1), 76–80.
38	Haring, L., Mõttus, R., Jaanson, P., Pilli, R., Mägi, K., & Maron, E.	Subjective Well-being under Neuroleptics Scales short form (SWN-K)	Patient	10	Haring, L., Mõttus, R., Jaanson, P., Pilli, R., Mägi, K., & Maron, E. (2013). Subjective Well-Being Under Neuroleptics Scale short form (SWN-K): reliability and validity in an Estonian speaking sample. *Annals of General Psychiatry*, *12*(1), 28.
39	Del Vecchio Good, M.-J., Smilkstein, G., Good, B. J., Shaffffer, T., & Arons, T.	APGAR Family Function Scale (Family APGAR)	Patient	5	Del Vecchio Good, M.-J., Smilkstein, G., Good, B. J., Shaffffer, T., & Arons, T. (1979). *The family APGAR index: A study of construct validity*.
40	World Health Organization	World Health Organization Quality of Life Questionnaire-Short Form (WHOQOL-BREF)	Patient	30	World Health Organization. (2012). *Programme on mental health: WHOQOL user manual, 2012 revision*. World Health Organization.
41	Awad, A. G., & Voruganti, L. N. P.	Body weight image and self-esteem evaluation questionnaire	Patient	3	Awad, A. G., & Voruganti, L. N. P. (2004). Body weight, image and self-esteem evaluation questionnaire: development and validation of a new scale. *Schizophrenia Research*, *70*(1), 63–67.

**Figure 1:**
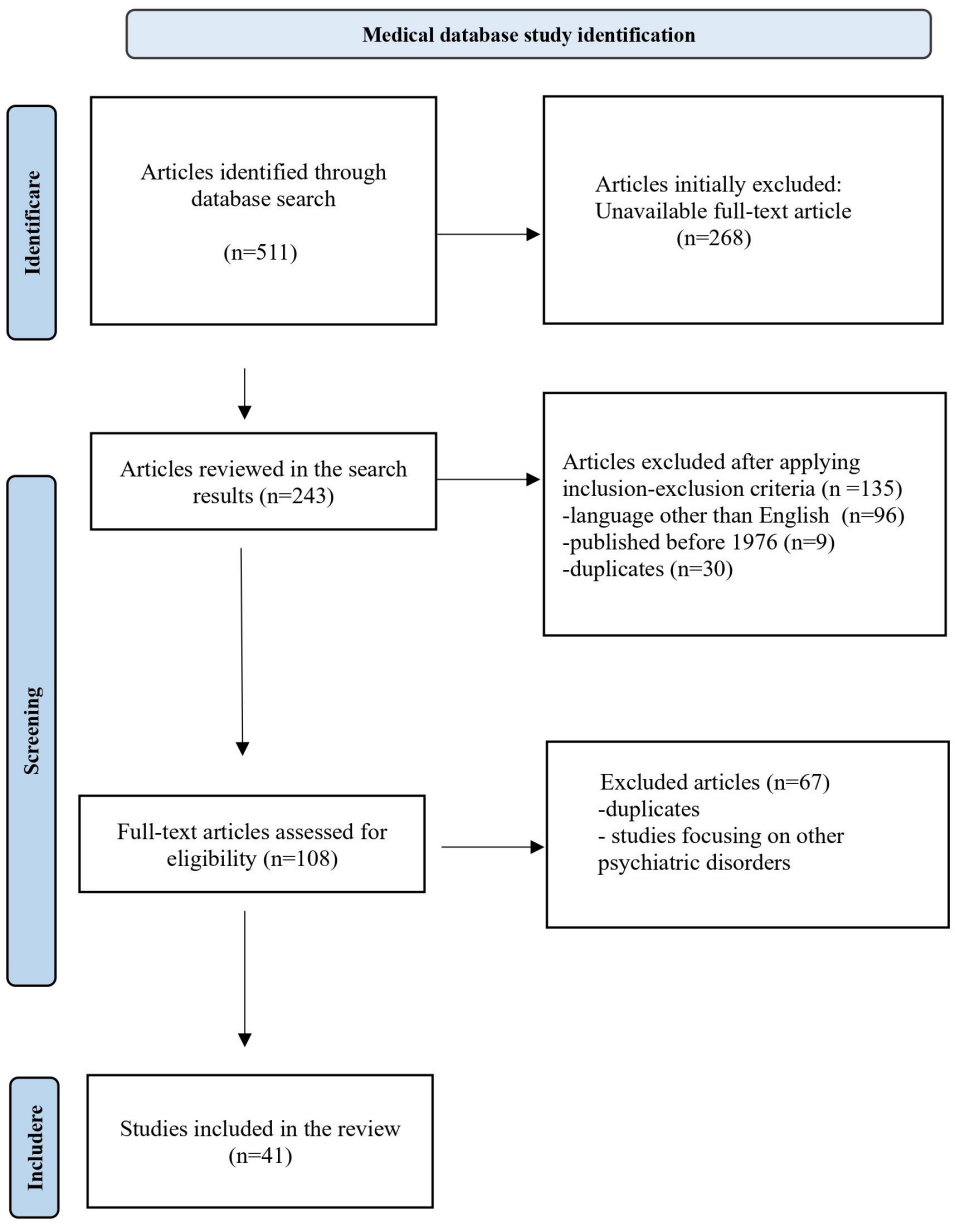
PRISMA diagram.

Relevant data were extracted using the PRISMA flow diagram (**Figure 1**), incorporating the above inclusion and exclusion criteria, removal of duplicates, and full-text screening. A total of 511 articles were initially identified: 304 from PubMed, 102 from NIH, 76 from Springer Nature Link, and 29 from Wiley Online Library. A total of 268 articles were excluded at the initial stage due to the lack of full-text availability. Another 135 were excluded: 96 due to language restrictions, 9 for being published before 1976, and 30 for being duplicates. After applying the inclusion criteria, 108 full-text articles were selected. Ultimately, after exclusion of the other 68 articles (duplicates or referring to other psychiatric pathologies), 41 articles were retained for inclusion in the final review (**Figure 1**).

## 4 Results

The scales and questionnaires selected for analysis are summarized in **Table 1**.

Notably, the *Independent Living Skills Survey* (24) exists in two formats—one completed by the patient and the other by a caregiver, each containing a different number of items and varying in administration time. Therefore, these two formats were treated as distinct instruments for the purpose of statistical analysis, resulting in a total of 42 scales included in the final evaluation.

**Figure 2:**
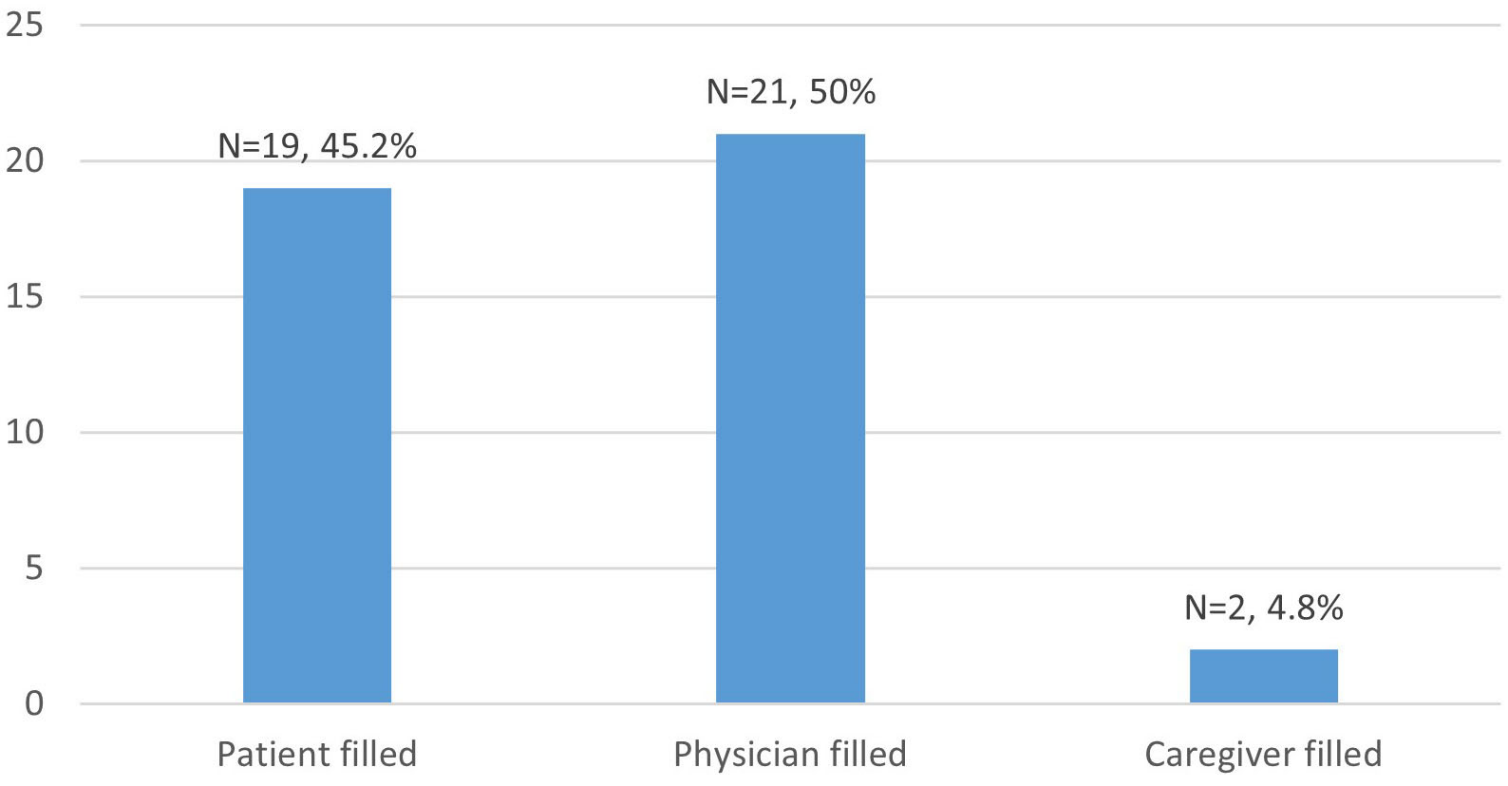
Questionnaire respondent.

Among the 42 instruments, 21 (50%) are clinician-administered and scored, 19 (45.2%) are self-assessment scales completed by the patient, and 2 (4.8%) are caregiver-completed questionnaires (**Figure 2**).

The average administration time across instruments was 19.59 minutes (SD ±14.88). The longest duration was reported for the *Modular System for Quality of Life* (MSQL-R) at 60 minutes (25), while the shortest duration—2 to 3 minutes—was noted for the self-administered *Body Weight, Image, and Self-Esteem Evaluation Questionnaire* (26).

The mean number of items per scale was 27.09 (SD ±24.54). The smallest number of items (5) was recorded in two scales: the *Performance-Based Skills Assessment – Brief Version (UPSA-B)* (27) and the *APGAR Family Function Scale* (28). The highest number of items was 105, in the Lancashire Quality of Life Profile (29).

**Figure 3:**
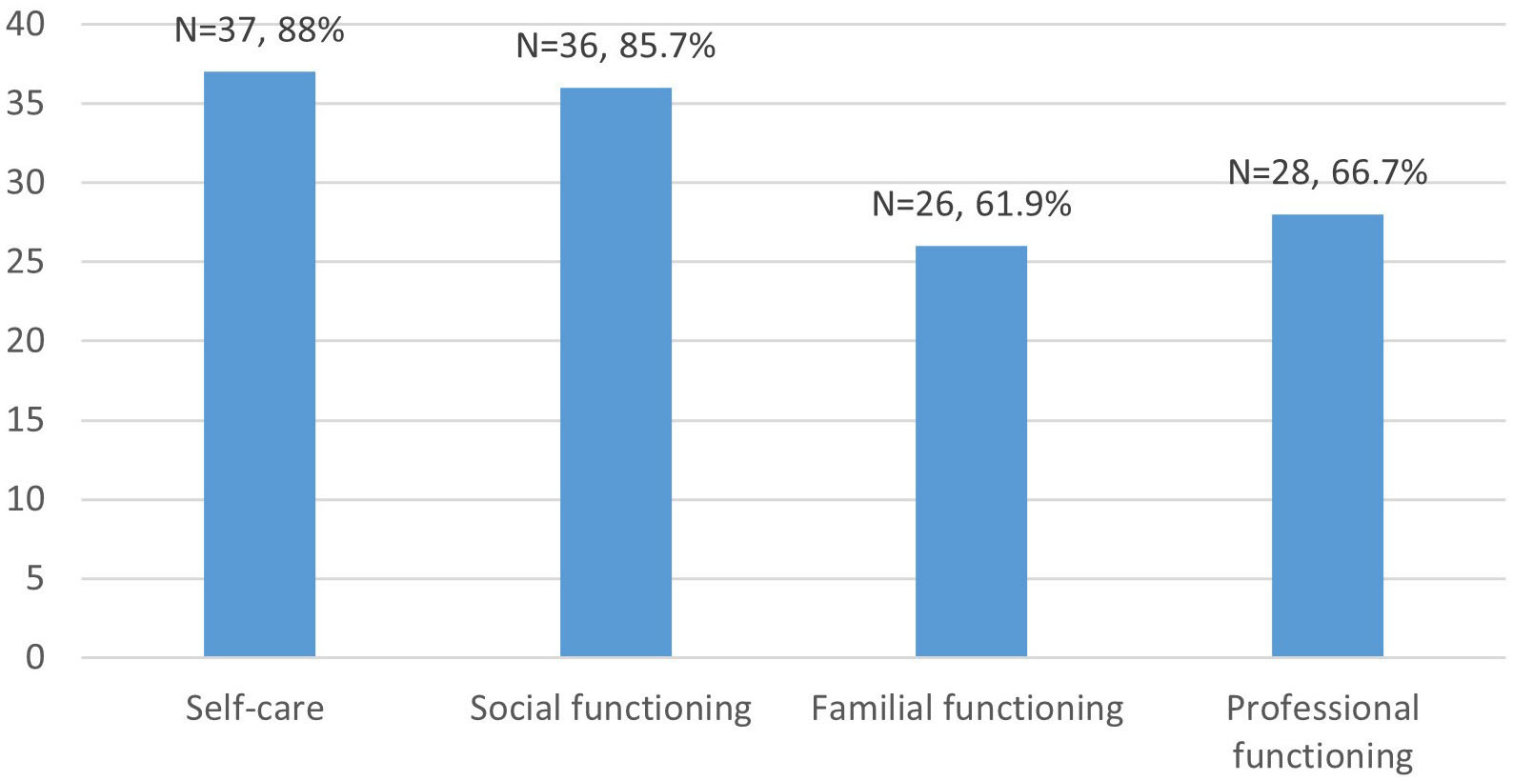
Investigational domains.

Of the 42 scales reviewed, 37 (88%) assessed the domain of self-care, 36 (85.7%) assessed social functioning, 26 (61.9%) assessed familial functioning, and 28 (66.7%) assessed occupational functioning (**Figure 3**).

Several scales also included items relating to physical or mental health within the self-care domain. Eleven scales queried general health status, six evaluated physical condition, and eleven included questions on maintaining a healthy diet. However, only one—*Body Weight, Image, and Self-Esteem Evaluation Questionnaire*—explicitly addressed the impact of weight gain secondary to psychotropic medication. Ten scales assessed sleep (qualitative and/or quantitative), and six assessed stress levels. Additional dimensions included decision-making capacity (14 scales), self-awareness and insight (9 scales), and self-confidence (9 scales). Only four scales contained items on fear and anxiety (“living in fear” constructs), three evaluated social anxiety, and ten assessed behavioral control.

In relation to treatment, 17 scales assessed treatment adherence as part of the self-care domain. Eighteen scales explored the use of medical services, but only nine explicitly addressed patients’ knowledge regarding their medication and its purpose.

## 5 Discussion

To date, a review of functionality assessment tools in schizophrenia has not been performed; existing reviews focus on negative or cognitive symptoms schizophrenia, and mostly target the instruments that are used in clinical trials. Our review included 42 scales assessing functionality in schizophrenia, regardless of the schizophrenia subtype or setting (daily evaluations in hospitals or practices, or clinical trials), categorized into clinician-administered, self-assessment, and caregiver-completed formats. Administration times and scale lengths varied widely. Most scales focused on self-care, social, familial, and occupational functioning. Additional items addressed physical and mental health aspects, including treatment adherence and medical service use, but few explored patients’ medication knowledge or anxiety-related dimensions. From a clinical and practical perspective, some features, like the assessment time, the rater type, or some particular domains, can make a tremendous difference, and are to be discussed here.

Historically, the primary aim of schizophrenia treatment was the remission of clinical symptoms. However, with the advancement of antipsychotic therapies, it became evident that many patients could achieve various degrees of social and occupational reintegration. As a result, the paradigm in schizophrenia treatment has shifted: functional recovery has emerged as a central therapeutic target. Functional improvement is now considered essential, beyond mere symptom relief, as it directly contributes to reintegration and quality of life. Accordingly, a comprehensive and accurate assessment of patient functionality has become an indispensable component in both clinical monitoring and treatment evaluation.

The time required to complete functionality scales is a crucial factor in clinical decision-making. As observed in other medical domains (30), lengthy and complex instruments may hinder routine use, especially in time-constrained outpatient settings. This concern has prompted the development of shorter versions of existing scales. For example, the WHOQOL scale initially included 236 items in its pilot version, later reduced to 100 (WHOQOL-100), and eventually to 26 items in the WHOQOL-BREF version (31).

Our findings confirm that the average administration time of functionality questionnaires is approximately 19.59 minutes (SD ±14.88), with some requiring up to 60 minutes. However, administration time may vary considerably, depending on the raters’ experience, or the patients’ psychopathological status. For instance, administration of the Functioning Assessment Short Test (FAST) may take from 15 minutes to 1 hour, depending on various factors pertaining to the rater or the patient (32).

Given the limited time allocated for clinical consultations, lengthy and complex instruments may be impractical for routine use. Moreover, since many of these scales are intended for repeated use to track changes over time, concise versions with clear wording and defined time frames are particularly valuable [e.g., WHOQOL-BREF (31) or S-QOL-18 (33)]. The advantage of applying the same scale in different moments resides in the fact that this might turn into an instrument for monitoring the evolution of the patients’ functionality, not only symptom relief, with a certain treatment.

Another critical consideration is the identity of the respondent. It is well documented that physicians often overestimate treatment adherence, regardless of the disorder (34). However, some studies (e.g., Olivares et al.) suggest that psychiatrists may have a more realistic view of adherence in schizophrenia patients (35). Conversely, other research has shown that knowledge of adherence status by the clinician does not significantly affect outcomes or resource utilization (36). Nevertheless, comprehensive psychopathological assessment remains the psychiatrist’s prerogative and should ideally be complemented, not replaced, by other perspectives. It is thus advisable for certain sections of functionality scales to be clinician-rated, while others may be completed by social workers or caregivers when appropriate.

Caregivers are a valuable source of information regarding the patient’s functioning in daily life. Randomized controlled trials often include caregiver input as part of outcome evaluations. Given their close and frequent contact with patients, they provide reliable observations regarding functioning across domains. However, only 3 out of the 42 reviewed scales (7.1%) include caregiver-completed versions, highlighting a missed opportunity to incorporate this critical perspective.

An important feature of the functionality assessment scales refers to the assessed domains. While most scales evaluate self-care, social, familial, and occupational domains, only a few take into consideration the patients’ treatment (adherence, knowledge). Treatment adherence plays a fundamental role in the life of a person with schizophrenia, who requires long-term pharmacological treatment, many times with multiple agents (37). As such, chronic treatment itself might be a determinant of both quality of life and functional outcomes. Antipsychotic treatment has been consistently associated with reduced frequency of psychotic episodes and hospitalizations (38). Emerging evidence also supports its role in prolonging life expectancy, improving quality of life, and preserving or even enhancing cognitive functioning, while potentially offering protection against serious somatic illnesses (39). Nonetheless, non-adherence remains a major challenge, with rates as high as 60% among schizophrenia patients (40). Non-adherence may be partial or complete and can include inconsistent dosing, incorrect timing, or skipped doses, making it difficult to quantify. Moreover, even partial non-adherence may result in an increased risk of relapse (41).

Despite its importance, few scales have been developed to assess treatment in schizophrenia. Existing tools predominantly focus on adherence [e.g., Medication Adherence Questionnaire (MAQ) (42) or Medication Adherence Rating Scale (MARS) (43)] or treatment satisfaction [e.g., the single-item Medication Satisfaction Questionnaire- MSQ (44)]. They rarely address patients’ knowledge of their treatment regimen, administration methods, potential side effects, or preventive strategies. Thus, a thorough evaluation of patients’ understanding of their pharmacological management is urgently needed.

An important aspect, regarding the self-assessed instruments, is that they have to consider that many schizophrenia patients have negative or cognitive symptoms, which might hinder the completion. Visual analog scales (VAS) could be a useful tool for assessing treatment adherence and knowledge. Simple, clearly worded questions—such as “How many times did you forget to take your medication last week?” or “On how many days last week did you take your medication exactly as prescribed?”—may facilitate accurate reporting.

Functionality scales applied only once, even if referencing a defined time period, offer only a cross-sectional snapshot of a patient’s life. In contrast, repeated application at different time points (e.g., at admission and follow-up post-discharge) allows for dynamic assessment of progress. Given that improved functioning may result from effective treatment, changes in functionality scores may serve as a proxy for treatment efficacy and disease evolution. Therefore, the routine clinical use of functionality assessment scales is of paramount importance.

Although symptoms significantly affect quality of life in schizophrenia, it is important to recognize that symptom-specific instruments already exist. Examples include PANSS, BPRS, and BACS for general, positive, negative, and cognitive symptoms (45–47), C-SSRS for suicidality (48), CDSS for depression (49), and SAES for anxiety in schizophrenia (50). Thus, the inclusion of symptom items in functionality scales such as FROGS (51) or MSQL-R (25) raises questions about redundancy and the risk of overlapping measurements.

For clinical use and applicability in clinical settings, no single “best” scale exists. Some instruments require specific training, which might limit their clinical everyday use. We suggest FAST (32) and SLOF (52) as being closest to the psychiatrists’ needs, in terms of assessment time (approximately 15 minutes each) and domains.

On the other hand, for research purposes, Long et al. found SFS (53) and PSP (23) to be most appropriate (54).

In recent years, interest in functional recovery among schizophrenia patients has grown substantially (55). An expert panel concluded that, despite its frequent use in practice, the concept remains poorly defined, and no existing scale is universally accepted as fully suitable for clinical needs (56). Gorwood and colleagues synthesized an expert panel recommendations on assessing functional recovery in schizophrenia. They identified the most relevant domains as: depression, aggressive behavior, social interaction, family functioning, education/employment, leisure activities, self-care, and sexual functioning (57). Searle et al. suggest that improvement in existing functionality assessment instruments is possible through digitalization (19). However, the evaluation of all the mentioned domains via technology might prove to be challenging.

Considering the existing literature, the type of assessment, physicians’ opinions, patient availability, and the duration, a functional assessment scale should most appropriately encompass the following domains:

- Self-care: defined as the individual’s ability to independently manage basic personal hygiene and grooming needs, such as regular bathing, dental hygiene, hair care, and appropriate attire selection, at a frequency that aligns with societal norms.
- Family functioning, referring to the capacity to maintain at least one consistent and meaningful interpersonal connection with a family member, characterized by reciprocal communication, emotional engagement, and participation in shared responsibilities or rituals (e.g., meals, caregiving, conflict resolution).
- Social functioning, denoting the individual’s ability to engage in at least one weekly interaction with a non-family member through in-person, telephonic, or digital communication. The interaction should demonstrate reciprocity, relevance, and social appropriateness, contributing to a sense of belonging or community integration.
- Occupational/educational functioning, involving the ability to sustain engagement in structured, goal-directed activities such as employment, academic study, or vocational training. Key indicators include punctuality, task completion, adherence to rules or curricula, and interaction with peers or supervisors in a functional manner.
- Sexual interest assesses the presence of age-appropriate sexual desire or romantic interest, which may manifest through thoughts, affective expression, or consensual engagement. It also includes the ability to form and sustain intimate relationships where relevant.
- Treatment knowledge encompasses awareness and understanding of one’s current therapeutic regimen, including the type and purpose of treatment (e.g., pharmacological or psychotherapeutic), prescribed dosage, potential side effects, and the importance of adherence. Evaluation may include verbal recall, informed decision-making, and demonstrated adherence, including respecting medical appointments for periodical prescriptions.
- Leisure activities domain reflects participation in voluntary activities undertaken for enjoyment or personal fulfillment. These may include hobbies, artistic expression, physical activity, or recreational pastimes, and should occur with some regularity and initiative.

These attributes should be rather stable over time and demonstrate low or no fluctuations for a patient to be considered functional.

We propose that an ideal scale should contain a minimal number of clearly worded questions and should specify a well-defined reference period. Also, the scale should be brief and easy to administer, and ideally should incorporate visual elements to facilitate understanding and application. A visual aid might have multiple benefits for schizophrenia patients, such as reducing cognitive load by simplifying abstract tasks, compensating for verbal limitations by providing visual context, supporting working memory by allowing external referencing rather than internal recall, and improving the reliability of patient self-report, particularly when insight is limited. Visual aids would be suitable for items such as leisure activities, social functioning, and treatment knowledge.

Such a scale would provide a comprehensive and clinically practical tool to monitor the functional trajectory of patients with schizophrenia, complementing symptom-based evaluations and supporting the overarching goal of recovery.

Like any review, this paper has its limitations. The most important is the lack of risk of bias assessment or evaluation of study quality. This paper emphasizes the role of functionality assessment in schizophrenia, and brings to clinicians’ and researchers’ attention a variety of instruments that can be applied for quantifying functional impairment in schizophrenia patients. At the same time, our results point out the imperfections of the existing instruments. By proposing some core features for an ideal instrument, this paper paves the way for future research and development of a functionality assessment scale in schizophrenia, which would meet the needs of both professionals and patients.

## 6 Conclusions

Functionality assessment scales in schizophrenia are absolutely necessary. They provide valuable information about the patient’s evolution under treatment. They should be easy to apply and capture the essential elements of functionality, being an indicator of the success or failure of the therapeutic plan.

## Data Availability

The original contributions presented in the study are included in the article/supplementary material. Further inquiries can be directed to the corresponding author.

## Glossary

GAF: Global Assessment of Functioning
PSP: Personal and Social Performance Scale
SOFAS: Social and Occupational Functioning Assessment Scale
GSDS-II: Groningen Social Disabilities Schedule-II
ILSS: Independent Living Skills Survey
LSP-16: Life Skills Profile (Abbreviated)
FAST: Functioning Assessment Short Test
MCAS: Multnomah Community Ability Scale
QOLS: Quality of Life Scale
QOL: Quality of Life
UPSA: University of California San Diego Performance-Based Skills Assessment
ILS: Independent Living Scales
TABS: Test of Adaptive Behavior in Schizophrenia
VRFCAT: Virtual Reality Functional Capacity Assessment Tool
WoRQ: Readiness for Work Questionnaire
PSRS: Psychosocial Remission in Schizophrenia Scale
UPSA-B: Performance-Based Skills Assessment – Brief Version
IADL: Instrumental Activities of Daily Living
SF-36: Short Form-36 Health Survey
QoLI: Quality of Life Interview
LQOLP: Lancashire Quality of Life Profile
SOFI: Schizophrenia Outcomes Functioning Interview
SLOF: Specific Level of Functioning Scale
FROGS: Functional Remission of General Schizophrenia
SCQ: Schizophrenia Caregiver Questionnaire
MSQL-R: Modular System for Quality of Life – Revised
SFQ: Social Functioning Questionnaire
Q-LES-Q-18: Quality of Life Enjoyment and Satisfaction Questionnaire – 18 items
CSQ: Communication Skills Questionnaire
SAS-M: Social Adjustment Scale – Modified
ERA: Self-Care Requisites Scale
S-QOL 18: Schizophrenia Quality of Life Questionnaire Short Form – Clinical Practice
SQLS-R4: Schizophrenia Quality of Life Scale – Revision 4
SubRAS: Subjective Recovery Assessment Scale
RAS-24: Recovery Assessment Scale – 24 items
RAS-8: Recovery Assessment Scale – 8 items
SLDS: Satisfaction with Life Domains Scale
SWN-K: Subjective Well-being under Neuroleptics – Short Form
APGAR: APGAR Family Function Scale
WHOQOL-BREF: World Health Organization Quality of Life – Short Form
BWISE: Body Weight, Image, and Self-Esteem Evaluation Questionnaire
PANSS: Positive and Negative Syndrome Scale
BPRS: Brief Psychiatric Rating Scale
BACS: Brief Assessment of Cognition in Schizophrenia
C-SSRS: Columbia-Suicide Severity Rating Scale
CDSS: Calgary Depression Scale for Schizophrenia
SAES: Schizophrenia Anxiety Evaluation Scale
MAQ: Medication Adherence Questionnaire
MARS: Medication Adherence Rating Scale
MSQ: Medication Satisfaction Questionnaire
VAS: Visual Analog Scale.
